# Predominantly epithelial-type synovial sarcoma with overwhelming neuroendocrine differentiation: a potential diagnostic pitfall

**DOI:** 10.1186/s13000-022-01243-2

**Published:** 2022-07-11

**Authors:** Ying Chen, Ning Zhou, Deyu Guo, Xiaodong Wang, Xin He, Yujuan Xu

**Affiliations:** 1 Department of Pathology, Guiqian International General Hospital, Guiyang, Guizhou Province China; 2 Department of Pathology, Sichuan Mianyang 404 Hospital, Mianyang, Sichuan Province China

**Keywords:** Epithelial-type, Synovial sarcoma, Neuroendocrine differentiation

## Abstract

**Background:**

Synovial sarcoma is an uncommon soft tissue tumor of soft tissue, characterized by a specific SS18 rearrangement. It generally manifests as a lesion composed of monomorphic spindle cells and sometimes shows variable epithelial differentiation. Epithelial-type synovial sarcoma is rare, and synovial sarcoma with overwhelming neuroendocrine differentiation has not been reported previously.

**Case presentation:**

Here, we present a case of a young man with an epithelial-type synovial sarcoma of the right leg that showed an overwhelming neuroendocrine differentiation. The diagnosis was confirmed by the detection of targeted fusion re-arrangement associated with synovial sarcoma.

**Conclusions:**

This study emphasizes the importance of molecular approaches in modern soft tissue pathology. Detecting the expression of neuroendocrine antigens in synovial sarcoma is a pre-requisite to avoid misdiagnosis of metastatic neuroendocrine tumor, malignant peripheral nerve sheath tumor with glandular differentiation, and carcinosarcoma.

## Background

Synovial sarcoma (SS) is a malignant mesenchymal neoplasm that occurs predominantly in older children and young adults. Histologically, there are two major subtypes (biphasic and monophasic spindle cell) and minor subtypes (monophasic epithelial and poorly differentiated). Here, we report an epithelial-type synovial sarcoma of the right leg showing an overwhelming neuroendocrine differentiation. To the best of our knowledge, this phenomenon has not been reported earlier.

## Case presentation

**Fig. 1:**
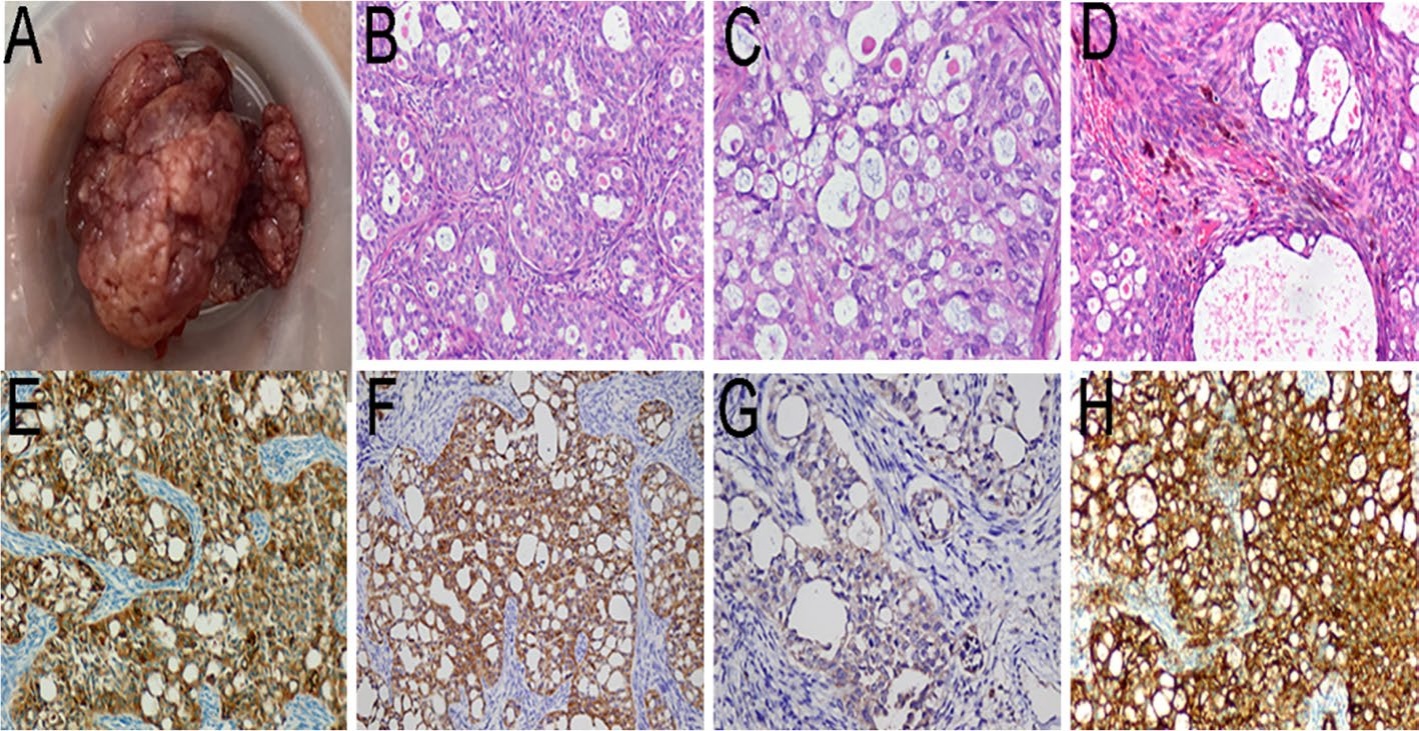
Photograph of the mass (**A**). Epithelial-like components showing cribriform or glandular pattern prominent throughout the neoplasm (**B** × 100). The epithelial cells are characterized by large, round or oval, vesicular nuclei and abundant pale-staining cytoplasm with indistinctly outlined cellular borders, and many goblet cells (**C** × 200). Focal areas of the neoplasm presented with well-developed spindle cell pattern (**D** × 200). Immunohistochemical studies demonstrated diffuse positive staining for EMA (**E **× 100), CK8/18 (**F** × 100), CgA (**G** × 100), and SYN (**H** × 100)

The patient was a 30-year-old Asian male with no significant past medical history. The right leg mass, which had been present for more than 1 year, had consciously and progressively enlarged with tenderness. This was surgically removed under the guidance of B-ultrasound. During the surgical procedure, after the subcutaneous tissue was cut, a tough mass of 30 mm × 30 mm × 20 mm with complete capsule, clear boundary, and good mobility was found in the muscle layer (Fig. 1A).

Pathological examination after the surgery at the referring hospital indicated a metastatic neuroendocrine tumor. On further diagnosis and treatment of the patient at our hospital, positron emission tomography/computed tomography (PET/CT) examination did not suggest any positive sign in other parts of the body.

**Fig. 2:**
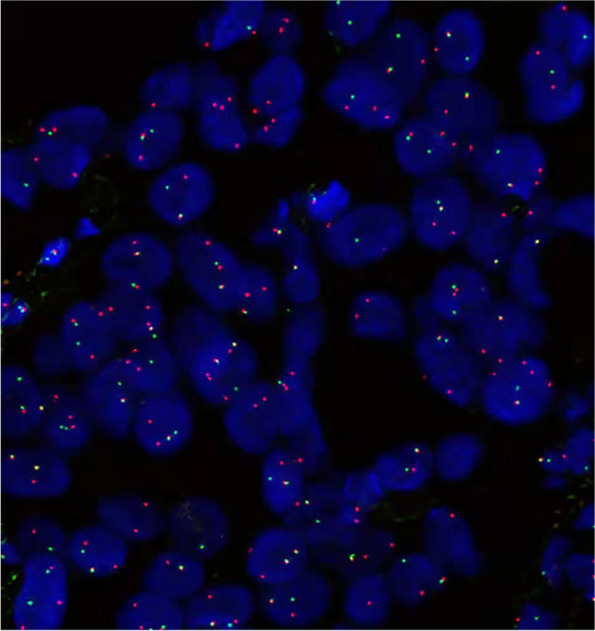
There was one pair of fusion signals and one pair of break-apart signals in 79% of the tumor cells

Pathological examination revealed that predominantly epithelial-like components presented a cribriform or glandular pattern throughout the neoplasm (Fig. 1 B,C). The epithelial cells were characterized by large, round or oval, vesicular nuclei and abundant pale-staining cytoplasm with indistinctly outlined cellular borders. They were arranged in solid cords and nests, with glandular and papillary architecture that contained mucus or homogeneous eosinophilic secretions. Focal areas of the neoplasm presented with well-developed spindle cell pattern composed of relatively uniform or slightly atypical surrounding and merging with the glandular structures (Fig. 1 D). Partial areas of gradual transition between the two components were present. The tumor was not necrotic, and mitotic figures were rare (3 mitoses per 50 high power fields). Moderate numbers of mast cells, areas of dense fibrosis, including bands of hyalinized collagen, and metaplastic bone in the stroma, were present. The immunohistochemical workup was notable for EMA (Fig. 1 E),CK8/18(Fig. 1 F), CD56, CgA (Fig. 1 G), SYN(Fig. 1 H), nestin, CD99, BCL-2, VIM, and H3K27me3 positivity in the epithelial cells. The results of all tests performed with site-specific markers are presented (Table 1). In addition, detection of SS18 break-apart was performed by fluorescence in situ hybridization analysis, and the tumor was showed SS18 gene rearrangement (Fig. 2). The final histological and immunohistochemical results confirmed the diagnosis of predominantly epithelial-type synovial sarcoma with overwhelming neuroendocrine differentiation. The patient recovered well and was recurrence for one year after follow-up observation.

**Table 1:** Immunohistochemical stains with interpretation of the results and technical data

Antibody	Result	Manufacturer	Species	Clone	Dilution	Stainer
CK8/18	**Positive**	**MXB**	**Mouse**	**MX035**	**Predilute**	**Ventana**
EMA	**Positive**	**MXB**	**Mouse**	**E29**	**Predilute**	**Ventana**
CD56	**Positive**	**MXB**	**Mouse**	**MX039**	**Predilute**	**Ventana**
CgA	**Positive**	**MXB**	**Mouse**	**MX018**	**Predilute**	**Ventana**
SYN	**Positive**	**MXB**	**Mouse**	**MX038**	**Predilute**	**Ventana**
Nestin	**Positive**	**MXB**	**Mouse**	**10C2**	**Predilute**	**Ventana**
BCL2	**Positive**	**MXB**	**Mouse**	**MX022**	**Predilute**	**Ventana**
CD99	**Positive**	**MXB**	**Mouse**	**O13**	**Predilute**	**Ventana**
CEA	**Negative**	**MXB**	**Mouse**	**MX068**	**Predilute**	**Ventana**
S100	**Negative**	**MXB**	**Mouse**	**4C4.9**	**Predilute**	**Ventana**
SOX10	**Negative**	**MXB**	**Rabbit**	**EP268**	**Predilute**	**Ventana**
Calponin	**Negative**	**MXB**	**Mouse**	**MX023**	**Predilute**	**Ventana**
Desmin	**Negative**	**MXB**	**Mouse**	**MX046**	**Predilute**	**Ventana**
VIM	**Positive**	**MXB**	**Mouse**	**MX034**	**Predilute**	**Ventana**
H3K27me3	**Positive**	**ZSB**	**Rabbit**	**RM175**	**Predilute**	**Ventana**
WT-1	**Negative**	**MXB**	**Mouse**	**MX012**	**Predilute**	**Ventana**
CDX2	**Negative**	**MXB**	**Mouse**	**MX024**	**Predilute**	**Ventana**
Ki67	**Index 5%**	**MXB**	**Rabbit**	**MXR002**	**Predilute**	**Ventana**

## Discussion and conclusions

Synovial sarcoma is a malignant mesenchymal neoplasm that occurs predominantly in older children and young adults. It has been shown to occur in almost any anatomical site and accounts for 5–10% of all soft tissue sarcomas. The average duration of symptoms is around 2–4 years, and some cases have recorded histories of 20 years [1, 2]. There is a slight male preponderance, with a male:female ratio of approximately 1.2:1. Histologically, there are two major subtypes (biphasic and monophasic spindle cell) and minor subtypes (monophasic epithelial and poorly differentiated). Predominantly epithelial-type synovial sarcoma is extremely rare, and few cases have been reported over the past four decades [3–5]. SS18 rearrangements or SS18-SSX can be detected in more than 95% of SS [6], specifically in all morphologic subtypes.

A challenging case of epithelial-predominant SS in the leg of a young man is presented in this study. The diagnosis was based on deep soft tissue site, patient age, lack of medical history, absence of visceral primary tumor, and the striking epithelial cell morphology. In this case, an exhaustive workup was performed. The epithelial cells were positive for CK8/18, EMA, CD99, BCL2, and VIM. Furthermore, our major findings revealed that cells of the tumor diffusely expressed neuroendocrine antigens, such as CD56, CgA, SYN, and nestin. These antigens are highly specific markers for neuroendocrine cells. This oservation has never been reported before. The staining pattern and clinical presentation were suggestive of a primary mesenchymal lesion, specifically synovial sarcoma, and molecular testing confirmed the diagnosis.

The differential diagnosis of predominantly epithelial-type synovial sarcoma with overwhelming neuroendocrine differentiation includes metastatic neuroendocrine tumor, malignant peripheral nerve sheath tumor (MPNST) with glandular differentiation, and carcinosarcoma [7]. In the present case, the tumor cells exhibited mild atypia, and the histological morphology was similar to that of neuroendocrine tumors. In addition, abnormal expression of neuroendocrine markers can lead to misdiagnosis. However, in this study, the patient did not have a previous history of neuroendocrine tumor, and a current primary tumor was not identified clinically or on imaging. A neuroendocrine tumor could not explain the presence of focal spindle tumor cells. Thus, the above information did not support a diagnosis of neuroendocrine tumors.

MPNSTs are soft tissue neoplasms with evidence of nerve sheath differentiation [8, 9]. They usually arise from peripheral nerves or from pre-existing benign nerve sheath neoplasms, often in patients with neurofibromatosis type 1 [10, 11]. MPNST with glandular differentiation is also a rare disease. The epithelial component can also be well demarcated and composed of prominent, well-formed glandular patterns. Considering that glandular cells can also stain positive for chromogranin and synaptophysin, without molecular studies, it was very difficult to distinguish between SS and MPNST. MPNST has been lacking in diagnostic immunohistochemical markers and specific genetic aberrations. Recent studies have shown that approximately half of MPNSTs lost their expression of H3K27 on immunohistochemical analysis [12], but this antigen was reactive in this case.

Additionally, the possibility of metastatic carcinosarcoma was considered, but was not favored due to the mild histological structure. Further workup for a primary tumor, including PET/ CT, was not indicated.

In conclusion, predominantly epithelial-type synovial sarcoma is rare, and synovial sarcoma with overwhelming neuroendocrine differentiation has not been reported previously. Pathologists should understand this phenomenon and avoid the diagnostic pitfall. The prognostic significance of prominent neuroendocrine differentiation in SS is unknown.

## Data Availability

All data generated or analyzed during the current study are included in this published article.

## Abbreviations

SS: Synovial sarcoma
PET/CT: Positron emission tomography/computed tomography
MPNST: Malignant peripheral nerve sheath tumor
